# Controlled Adhesion and Growth of Long Term Glial and Neuronal Cultures on Parylene-C

**DOI:** 10.1371/journal.pone.0025411

**Published:** 2011-09-22

**Authors:** Evangelos Delivopoulos, Alan F. Murray

**Affiliations:** 1 Nanoscience Centre Department of Engineering, The University of Cambridge, Cambridge, Cambridgeshire, United Kingdom; 2 School of Engineering and Electronics, Institute for Integrated Micro and Nano Systems, The University of Edinburgh, Edinburgh, Midlothian, United Kingdom; Michigan State University, United States of America

## Abstract

This paper explores the long term development of networks of glia and neurons on patterns of Parylene-C on a SiO_2_ substrate. We harvested glia and neurons from the Sprague-Dawley (P1–P7) rat hippocampus and utilized an established cell patterning technique in order to investigate cellular migration, over the course of 3 weeks. This work demonstrates that uncontrolled glial mitosis gradually disrupts cellular patterns that are established early during culture. This effect is not attributed to a loss of protein from the Parylene-C surface, as nitrogen levels on the substrate remain stable over 3 weeks. The inclusion of the anti-mitotic cytarabine (Ara-C) in the culture medium moderates glial division and thus, adequately preserves initial glial and neuronal conformity to underlying patterns. Neuronal apoptosis, often associated with the use of Ara-C, is mitigated by the addition of brain derived neurotrophic factor (BDNF). We believe that with the right combination of glial inhibitors and neuronal promoters, the Parylene-C based cell patterning method can generate structured, active neural networks that can be sustained and investigated over extended periods of time. To our knowledge this is the first report on the concurrent application of Ara-C and BDNF on patterned cell cultures.

## Introduction

Patterned cell cultures have been receiving increasing interest and scrutiny from the scientific community. They offer insight into cellular development, while revealing the processes underlying cell adhesion, growth and proliferation. On the network scale, patterned neural networks can offer valuable understanding of diseases, such as epilepsy and stroke, while providing a platform for the development and screening of drugs. Established techniques in the field include micro-contact stamping [1] and microfluidics [2]. Thin film micro-contact printing [3], and use of PDMS or parylene-C stencils [4], [5] are recently developed methods. Patterned neuronal cultures [6] are constantly employed in multi electrode arrays (MEA) [7], [8] and electrophysiological recordings, not only to understand the *in vivo* development of such networks, but also to investigate how information is processed in the brain [9]. Lately, it has been demonstrated that neural processes can be confined in and guided through tubular nanomembrane arrays, that could potentially mimic the myelin sheath [10].

A number of design parameters are optimized during a cell patterning experiment. These include media composition, plating density of the cells, nature of the tissue (cell line, dissociated primary cells, tissue slices), chemistry and topography of the substrate and finally the *in vitro* incubation period of the culture. Brain cell patterning is particularly challenging, as glia respond weakly to chemical and topographical cues and proliferate throughout the duration of the culture, to form a “glial carpet” over the entire substrate [7]. This tends to gradually distract neurons off the underlying pattern, as neurons usually co-localize with glia [11]. It has been shown that spontaneous activity in cultured neural networks may emerge as early as 10–12 DIV [12], [13]. However, such activity in patterned neural networks usually becomes significant and recordable after the third week *in vitro* [14]. Therefore, it is essential to preserve neuronal compliance to patterns for long periods of time, when attempting to record spontaneous activity from a patterned neural network.

We recently presented a cell patterning technique based on the bio-polymer Parylene-C, often used as an encapsulant for microelectrodes and probes [15], [16] and known for its biocompatibility [17], [18]. Glia and neurons from the rat hippocampus were cultured on Parylene-C stripes that were photolithographically patterned on a SiO_2_ background [19]. This method of patterning is easy and simple to use, as it requires a single serum immersion step of the substrate after fabrication. It is also highly versatile, as the properties of Parylene-C can be altered via UV irradiation, modifying the permittivity of the substrate to neurons and glia [20]. Moreover, the thickness of Parylene-C can be reduced down to 10 nm rendering this an ideal encapsulant of passive electrodes in capacitive coupling recordings [21]. Finally, our patterning technique can be applied to other cell types, such as the human teratocarcinoma cell line [22], to a single cell resolution [23].

One of the current limitations of our technique is that even though at 7 DIV both glia and neurons exhibit high conformity to underlying Parylene-C stripes, at 14 and 21 DIV cellular compliance gradually deteriorates and is eventually lost. Usually, non-permissive coatings such as polyethylene glycol (PEG) and alkylsilanes are employed to restrict neuronal growth off patterns [6], [24]. However, such “cytophobic” layers are not always stable long term and degrade, due to oxidation or chain cleavage. Furthermore, the success of this approach depends heavily on the careful selection of an appropriate chemistry, which ensures the self-assembly of the chosen molecules on the correct substrates and prevents the disruption of previously assembled molecules. On the other hand, during the first week of culture, our cell patterning technique provides a high contrast between adhesive and non-adhesive substrates, without the need of additional complicated steps.

In our current paper, we explore how the proliferation of glia (or lack thereof) affects neuronal network development on our engineered substrates. We present evidence illustrating that glial mitosis is responsible for both neuronal and glial off-pattern migration. XPS characterization of our substrates at 7, 14 and 21 DIV revealed that the loss of organization is not due to protein desorption from the Parylene-C or SiO_2_, as nitrogen levels remain stable over the course of 3 weeks. We limited glial division with the use of cytosine arabinoside [25], [26] (AraC) and thus preserved neuronal and glial conformity to Parylene-C stripes. Neuronal apoptosis, which is often induced by AraC was prevented with the inclusion of brain derived neurotrophic factor [27] (BDNF). The challenge in this approach exists in finding a compromise between AraC toxicity and BDNF mediated glial enhancement [28]. We are currently recording calcium transients from glial and neuronal cultures at 14 and 21 DIV [29], [30] thus, establishing that these cultures can be viable tools in electrophysiological and neuroscientific studies (unpublished data).

## Materials and Methods

### 1. Ethics Statement

All protocols and procedures involving animals were carried out with the approval of the Home Office and in strict adherence to the Animals (Scientific Procedures) Act 1986. Our study was also approved by the Ethical Review Committee of The University of Edinburgh. Project and personal license numbers are withheld by the Home Office and can be provided upon specific request.

### 2. Substrates: Fabrication, Cleaning, Sterilisation and Activation

**Figure 1:**
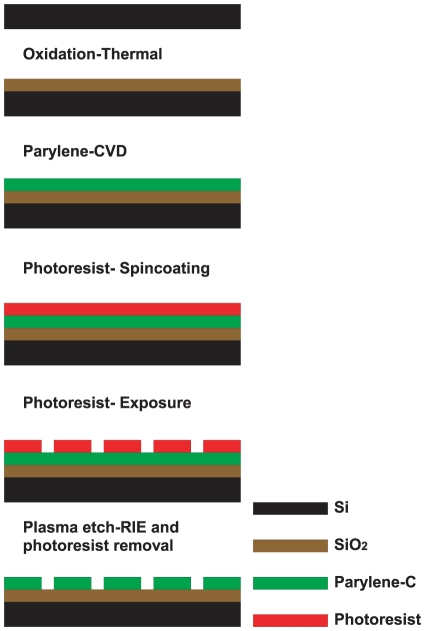
Process flow of parylene-C pattern fabrication procedure.

A 100 nm layer of Parylene-C was deposited at room temperature (1.298 nm per mg of dimer) on a 200 nm layer of SiO_2_ (H_2_ 1.88sccm and O_2_ 1.25sccm, 950°C for 40 minutes) grown on silicon (verification via inspection with Nanospec). Wafers were then coated with hexamethyldisilazane (HMDS) and positive photo-resist Rohm & Hass SPR350-1.2 (1 µm theoretical thickness), followed by a 60 second soft bake at 90°C. We used an Optimetrix 8605 5× reduction stepper together with a previously created mask to print stripe patterns on each wafer. For uniform surfaces (no printed pattern), wafers either remained unexposed (uniform parylene), or were flood exposed (uniform SiO_2_). Wafers were developed in Microchem MF-26A, after a 60 second post exposure bake at 110°C. No hard bake was performed. Unprotected parylene was etched off in the Plasma therm (90 seconds, 50mTorr chamber pressure, 50 sccm O_2_, 500W RF power, etch rate is approximately 100 nm/min) to reveal the SiO_2_ underneath. Complete etching was verified with the Nanospec. Residual photo-resist was removed with acetone. Wafers were cut with a DISCO DAD 800 Dicing Saw (spindle speed 30000 rpm, feed speed 7 mm/sec), rinsed with distilled ionized (DI) water and blown dry with Nitrogen. The fabrication process flow is also illustrated in Figure 1.

We tested four different stripe patterns that were 2 mm long and 10 µm, 20 µm, 30 µm and 40 µm wide. Data from different pattern geometries were analyzed together. Equal N numbers of different geometries were tested.

Individual substrates were cleaned in piranha acid (30% H_2_O_2_, 98% H_2_SO_4_ in a 5∶3 ratio), sterilized in penicillin/streptomycin for 1 hour and activated by a 3-hour immersion in horse serum (GIBCO®, New Zealand).

Glass coverslips cleaned in ethanol and immersed in 0.5 ml of poly-D-lysine solution (50 µg/ml) overnight were cultured in order to verify that the neuronal population within each harvest was adequately large and morphologically healthy.

### 3. Cell Cultures and Staining

We adapted the gradient cell isolation protocol presented by Brewer. We harvested and mechanically dissociated hippocampal cells from P1–P7 (postnatal) Sprague-Dawley rats. Cells were plated at 90 cells/mm^2^ in Neurobasal/B27 medium containing L-glutamine (0.5 mM), penicillin-streptomycin (125units/ml) and bFGF (10 ng/ml). Cultures were incubated at 37°C and 5% CO_2_ for 1, 2 and 3 weeks. Old media was exchanged with fresh media at 3 days after plating. After the first week media was exchanged every 2 days, with 5 µM cytosine arabinoside (AraC) being added in every other exchange to control glia division. In some experimental groups, we also added 10 ng/ml or 20 ng/ml brain derived neurotrophic factor (BDNF) with the AraC, to promote neuronal survivability and health.

After the incubation period, the cultures were rinsed once in a Tris(hydroxymethyl)aminomethane buffered saline solution (Tris) for 5 minutes, fixed in a 4% para-formaldehyde solution for 30 minutes and washed once in fresh Tris solution for 5 minutes. Fixed cells were then treated with 0.2% Triton-X100 solution for 15 minutes, to permeate cell membranes and immersed in donkey serum solution made in 0.2% Triton-X100 for 1 hour, to block against non specific binding. Primary antibodies (mouse anti-beta-tubulin from Sigma© at 1∶500 dilution for neuronal specific labeling and goat anti-GFAP from Sigma© at 1∶50 dilution for glia (astrocyte) specific labelling) were applied in blocking solution for one hour at room temperature (alternatively the antibodies were applied overnight at 4°C). Cells were rinsed twice in 0.2% Triton-X100 solution for 5 minutes. Secondary antibodies (donkey anti-mouse 594 Alexa Fluor™ from Molecular Probes at 1∶500 dilution and donkey anti-goat 488 Alexa Fluor™ from Molecular Probes at 1∶50 dilution) were applied in blocking solution as well. Cells were rinsed twice in Tris solution and nuclei were stained with TO-PRO 3 (made in Tris solution, 1∶5000 dilution) for 20 minutes. Finally, surfaces were rinsed once in Tris solution and once in distilled water and then mounted on glass slides with Mowoil mounting agent.

We used a Leica Confocal Microscope (Laser: Leica Lasertechnik Gmbh, Type TCS NT, Scan Unit: Leica DMRE, Leica TCS NT, Type:020-525.756, UV lamp: Ebq 100 isolated-L/131-26B, Prof. class IP 20) to take ×5 and ×20 (Leica Germany HC PL FLUOTAR lenses) images covering areas of 4 mm^2^ and 0.25 mm^2^ respectively. We imaged at the excitation wavelengths of the antibodies (590 nm, 495 nm and 642 nm) and the reflection of each pattern.

### 4. Statistical Analysis

The conformity of the cells to the parylene stripes was assessed by two indexes. In one, we compared the nuclear density of glia and neurons on the parylene to the respective density on the SiO_2_ background. In the other, we contrasted the green, red and black pixel intensity on the parylene stripes to the respective intensity on SiO_2_ background.

#### 4.1 Nuclear Conformity Index

We scored (*ImageJ*) cell nuclei on the parylene stripes and compared them to those on SiO_2_. Nuclei lying on the borders of stripes were considered to be on the parylene. The image (×20 lens) covered a 500 µm×500 µm square area at the center of the pattern and included 3 parylene stripes and 2 SiO_2_ backgrounds. We averaged and normalized with regards to surface area, the number of nuclei on the parylene stripes and did the same for the SiO_2_ backgrounds. The **nuclear conformity index** (NuCI) is the ratio of the parylene average to the SiO_2_ average. Two way ANOVA and correlation tests were performed on NuCI derived from different images.

#### 4.2 Glial and Neuronal Conformity Indexes

We individually selected (Leica Lite software) each parylene stripe and SiO_2_ background and calculated the histograms of the pixel intensity for both green (glia) and red (neurons) channels.

We thresholded the image corresponding to the histograms, aiming to include the maximum biological data possible (axons, dendrites and cell somata), without adding artifactual noise. The histogram area above the threshold value was considered as the quantity of glial (green) or neuronal (red) pixels present, while the area below was taken as the amount of void space (black pixels). Pixels were normalized with regards to surface area by dividing with the total number of pixels on each stripe. For example:
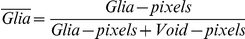
After acquiring the normalized glial, neuronal and void pixels of each parylene stripe *i* and SiO_2_ background *j*, we averaged across all parylene stripes and SiO_2_ backgrounds:
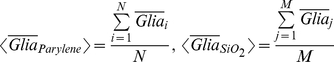
The **glial conformity index** (GCI) is the ratio of the average normalized glial pixels on the parylene stripes to the average normalized glial pixels on the SiO_2_ backgrounds. The **neuronal conformity index** (NeCI) is produced similarly. Likewise, two **void indexes** one corresponding to glia (VIg) and one to neurons (VIn) were also calculated, to indicate the overall number of glia and neurons present on the patterns respectively.
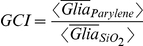
Values of GCI and NeCI run from 1 (random culture) to ∞ (perfectly patterned culture), while values of VI range from 1 (random culture) to 0 (perfectly patterned culture). If neurons and glia were to adhere to SiO_2_ and avoid parylene, glial and neuronal conformity indexes would range from 1 (random culture) to 0 (perfectly reversely patterned culture), while void indexes would run from 1 (random culture) to ∞ (perfectly reversely patterned culture).

Two way ANOVA and correlation tests were performed on indexes derived from different images.

#### 4.3 Polar plots

Images of patterned cultures were plotted as data points in a polar coordinate system. The *GCI_i_* and VIg_i_ of each image *i* indicated the radial coordinate *ρ_i_* and azimuthian angle *θ_i_* of each data point *i*. Therefore, data points further from the center signify images with high glial conformity to the parylene stripes. Void indexes were expressed in degrees via the following transformation:
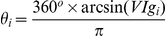
As *VIg_i_* values range from 0 to 1, *θ_i_* values range from 0° to 180° degrees. Therefore, data points with large polar angles indicate images with low number of glia.

In a few instances where the black ratio ranged from 1 to ∞ the following transformation was adopted:
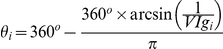
A polar scatter with NeCI and corresponding VIn was also plotted to highlight neuronal conformity and mass on the patterns.

### 5. Surface Analysis with XPS

Uniform parylene-C and SiO_2_ surfaces were cleaned in piranha acid and immersed overnight either in horse serum or distilled sterile water (controls). After the overnight incubation, control and horse serum treated chips were rinsed twice with distilled sterile water, placed inside sterile 24-well plates individually and taken to a VG Scientific, Sigma Probe XPS machine, inside a vacuum desiccator in order to prevent contamination of the surfaces. One general (zoom out, low resolution) and 4 element specific (zoom in, high resolution) XPS scans were executed on each uniform surface. In the zoom out scan, 7 passes were performed with the pass energy of the beam at 80 eV and dwell time at 40 msec. In the elemental scans the pass energy was 20 eV while the dwell time was again 40 msec. The number of passes in the high resolution scans ranged between 60 and 100, depending on the element and the quality of the spectrum. In all cases, the radius, or spot size, of the beam was 400 µm while the pressure in the vacuum chamber was always below 2*10^−8^ Torr. The energy of the X-ray source was (AlKa) 1486.6 eV. Data was collected as percentages of the contribution of each element in the surface composition.

#### 5.1 Protein desorption with Sodium Dodecyl Sulfate (SDS)

Uniform parylene-C and SiO_2_ surfaces were cleaned in piranha acid and immersed overnight either in horse serum or distilled sterile water (No HS controls). Serum immersed samples were then rinsed with SDS once (group 1) or thrice (group 2). The first, second and third SDS rinses lasted for 150, 90 and 60 minutes respectively. The surfaces were rinsed with distilled sterile water in between SDS rinses, while fresh SDS was used for each rinse. A few serum immersed samples (HS controls) and all the water immersed controls (No HS) were not rinsed with SDS, but directly scanned with XPS, in order to establish the average minimum and maximum levels of nitrogen on the substrates. All surfaces were rinsed twice in distilled sterile water before being analyzed with XPS as explained above.

## Results

### 1. Effect of AraC on long term glial and neuronal patterning

We conducted two independent experiments to examine the effect of glial division on the cellular conformity to the parylene-C patterns over the course of three weeks. We also investigated whether the addition of AraC mitigated glial division and preserved glial and neuronal conformity. All surfaces were initially immersed in horse serum for 3 hours. The control group was cultured in normal media, while the AraC group was cultured in media supplemented with cytarabine. The average plating density was 100 cells/mm^2^. Data from different experiments was processed together in two-way ANOVAs. Overall N numbers are given in Table 1.

**Table 1:** N numbers for experiments 1–4.

Experiment	Group	7 DIV	14 DIV	21 DIV
1 & 2	Control	21	8	8
	AraC	–	28	24
3 & 4	Control	24	16	16
	AraC	–	12	12
	AraC+BDNF	–	12	12
	AraC+BDNF+	–	12	14

**Figure 2:**
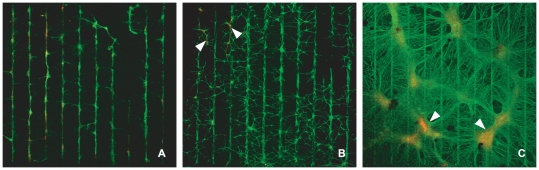
Culture examples of glia and neurons over a period of three weeks. (A) Culture fixed and stained at 7DIV (20 µm stripe thickness). (B) Culture fixed and stained at 14DIV (30 µm stripe thickness). (C) Culture fixed and stained at 21 DIV (20 µm stripe thickness). Glial and neuronal conformity progressively deteriorates from 7 to 21DIV. All images at ×5 magnification.

**Figure 3:**
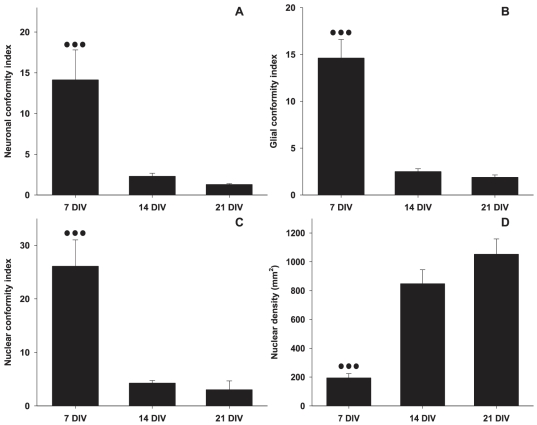
Neuronal, glial and nuclear conformity to parylene-C stripes on control substrates at 7, 14 and 21 DIV. (A) Neuronal conformity. (B) Glial conformity. (C) Nuclear conformity. (D) Nuclear density over the course of 3 weeks. Error bars represent the Standard Error of the Means (SEM). Three dots represent statistical significance of P≤0.001.

The sequence of culture images in Figure 2 demonstrates the degradation of neuronal and glial conformity to the underlying parylene-C patterns over the course of 3 weeks. At the end of the first week (Figure 2a, 7DIV), both glia and neurons conform to the parylene-C stripes as in previous patterning experiments [19]. There is minor bridging of stripes by glial processes however, cells remain predominantly on the parylene-C. By the end of the second week (Figure 2b, 14DIV) glia spread extensively across the SiO_2_ background. Neurons are distracted off the parylene-C and are following glial processes on the SiO_2_ (arrows in Figure 2b). However, structure is still evident in these cultures. At the end of the third week (Figure 2c), glia have formed large clusters and cover the entire surface with processes. Neurons, are entwined within the glial “carpet” and aggregate into large “neurospheres” that are located at the center of the glial clusters (arrows in Figure 2c). This decline in the quality of cell patterns over time is expressed by the statistical analysis. Figure 3 reveals a statistically significant decrease in glial and neuronal conformity across three weeks. No statistically significant differences were found between patterns of different stripe widths (P = 0.895, P = 0.743 for glia and neurons respectively).

**Figure 4:**
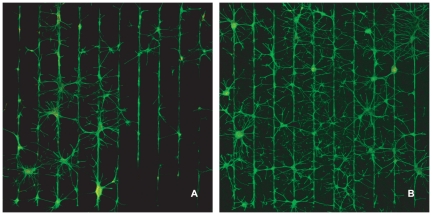
The addition of AraC preserves glial and neuronal conformity at 14DIV and 21DIV. (A) Culture example of glia and neurons at 14DIV (20 µm stripe thickness.). (B) Culture example of glia and neurons at 21 DIV (10 µm stripe thickness.). All images at ×5 magnification.

**Figure 5:**
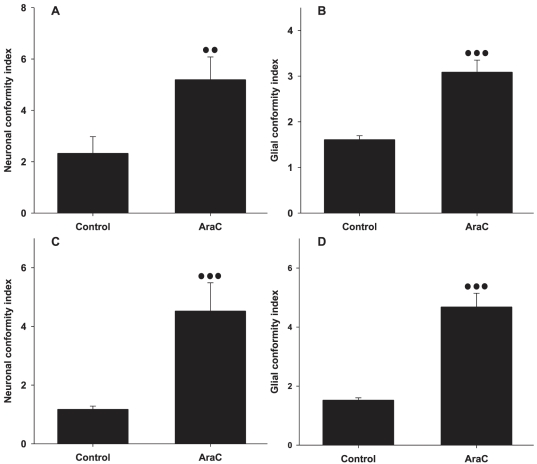
Neuronal and glial conformity to parylene-C stripes in control and AraC treated cultures. (A) Neuronal conformity at 14DIV. (B) Glial conformity at 14DIV. (C) Neuronal conformity at 21DIV. (D) Glial conformity at 21DIV. Error bars represent the Standard Error of the Means (SEM). Three and two dots represent statistical significance of P≤0.001 and P≤0.01 respectively.

The addition of AraC however, mitigates the loss of patterning at weeks 2 and 3. In the sequence of images in Figure 4 cells were cultured in AraC complemented media. We can clearly see that the spreading of glial and neuronal processes onto the SiO_2_ is not as predominant as in the control images of the second and third weeks. This is confirmed by the statistical analysis summarized in the bar graphs of Figure 5. Indeed, the neuronal and glial conformity indexes in the AraC and control groups differ by a factor of 2 at the end of the second week. This difference increases slightly at the end of the third week. Again, no statistically significant differences were found between patterns of different stripe widths (P = 0.977, P = 0.276 for 14 and 21DIV respectively).

### 2. Application of BDNF in conjunction with AraC treatment of patterned glial and neuronal cultures

The addition of AraC in the culture media is often detrimental to neuronal survival and health. We noticed decreased presence of neurons in AraC treated cultures after 2 weeks. After 3 weeks few neurons were present. In order to encourage neuronal growth and mitigate the adverse effects of AraC, we decided to apply BDNF concurrently with cytarabine.

We performed two independent experiments to observe the effect of BDNF on neuronal survivability over the course of three weeks. In two experimental groups we applied 10 ng/ml and 20 ng/ml of BDNF respectively, together with 5 µM AraC. We also included a control group with only 5 µM AraC and another control group which was cultured just in the usual media. All surfaces were initially immersed in horse serum for 3 hours. The average plating density was 85 cells/mm^2^. Data from different experiments was processed together in two-way ANOVAs. Overall N numbers are given in Table 1.

**Figure 6:**
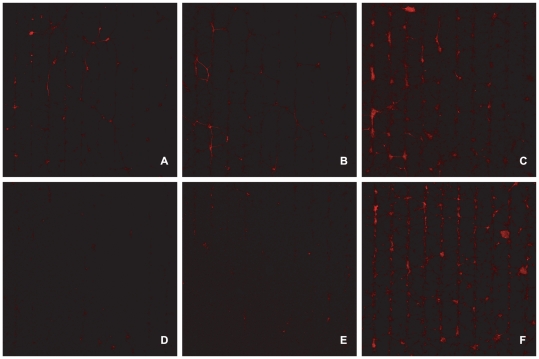
Neuronal presence in cultures treated with AraC only and cultures treated with AraC and BDNF, over a period of three weeks. (A) Culture treated with AraC only, fixed and stained at 14DIV (10 µm stripe thickness). (B) Culture treated with AraC and BDNF, fixed and stained at 14DIV (40 µm stripe thickness.). (C) Culture treated with AraC and a double dose of BDNF, fixed and stained at 14DIV (40 µm stripe thickness.). (D) Culture treated with AraC only, fixed and stained at 21DIV (40 µm stripe thickness.). (E) Culture treated with AraC and BDNF, fixed and stained at 21DIV (40 µm stripe thickness.). (F) Culture treated with AraC and a double dose of BDNF, fixed and stained at 21DIV (40 µm stripe thickness.). The “AraC only” group (A,D) has substantially fewer neurons than the “AraC&BDNF” group (B,E) and the “AraC&BDNF+” group (double dose of BDNF) (C,F). Also, at three weeks, few neurons are present on AraC and AraC&BDNF substrates (D,E). All images at ×5 magnification.

The culture images of Figure 6 illustrate the difference in neuronal health and presence between the AraC group and groups supplemented with BDNF. At 14DIV (Figure 6a–6c) and 21DIV (Figure 6d–6f) the group supplemented with the high dose of BDNF (Figure 6c and 6f) is superior to the other two.

**Figure 7:**
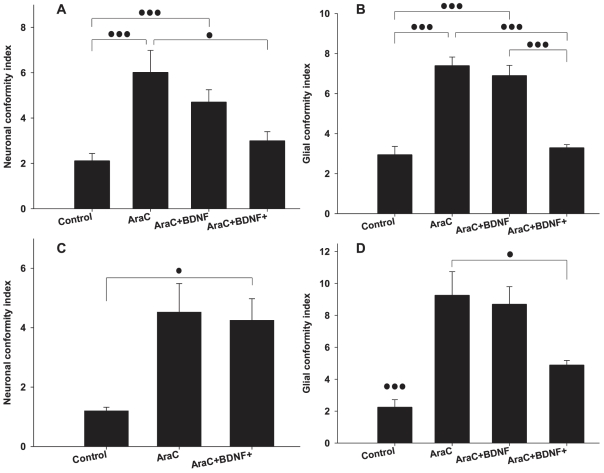
Neuronal and glial conformity to parylene-C stripes in control, AraC, AraC+BDNF and AraC+BDNF+ treated cultures. (A) Neuronal conformity at 14DIV. (B) Glial conformity at 14DIV. (C) Neuronal conformity at 21DIV. (D) Glial conformity at 21DIV. Error bars represent the Standard Error of the Means (SEM). One and three dots represent statistical significance of P≤0.05 and P≤0.001 respectively.

In Figure 7 we provide the statistical analysis of the glial and neuronal conformity indexes. We notice a decrease in both indexes that is associated with the presence of BDNF. However, indexes always remain higher than the control group, even when a high BDNF dose was added. A neuronal conformity index is not provided for the AraC+BDNF group at 21DIV, as the neuronal population was low in these substrates. The width of the stripes did not affect glial or neuronal conformity significantly (P = 0.392, P = 0.160 for glia and neurons at 14DIV and P = 0.624, P = 0.705 for glia and neurons at 21DIV).

### 3. Analysing the surface protein load using XPS

We performed two independent experiments in order to understand the nature of protein adsorption on the parylene-C and SiO_2_ substrates.

In our first experiment we immersed uniform parylene-C and SiO_2_ surfaces in horse serum overnight (controls were immersed in distilled sterile water instead) and then incubated them in normal culture media (Neurobasal-A), under normal culturing conditions (37°C, 5% CO_2_, frequent changing of media). We then monitored nitrogen levels over the course of 3 weeks by scanning our samples with XPS at different time points. We also included a group that was scanned immediately after the overnight incubation (horse serum or distilled sterile water), to attain starting nitrogen levels, for comparison.

**Figure 8:**
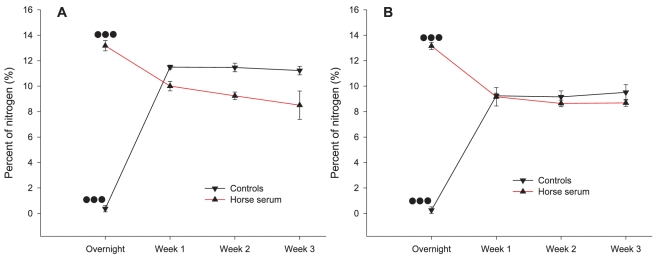
Nitrogen levels on parylene-C and SiO_2_ over a period of three weeks. (A) Parylene-C. (B) SiO_2_. On control samples (parylene-C and SiO_2_), overnight levels of nitrogen are neglible, but rise after one week due to the deposition of proteins present in the media. On horse serum treated samples (parylene-C and SiO_2_), nitrogen presence peaks after the overnight treatment, decreases slightly during the first week and stabilizes during the second and third weeks. Three dots represent statistical significance of P≤0.001.

Two way ANOVA tests revealed that overnight levels of nitrogen on water immersed parylene-C (0.372%±0.247%, n = 5) were significantly different from levels of week 1 (11.498%±0.155%, n = 5), week 2 (11.468%±0.339%, n = 5) and week 3(11.228%±0.338%, n = 4) (P≤0.001 in all cases). Similarly, on horse serum immersed parylene-C, overnight nitrogen levels (13.176%±0.401%, n = 7) were statistically different from average nitrogen levels of week 1 (10.009%±0.376%, n = 8), week 2 (9.236%±0.298%, n = 10) and week 3 (8.508%±1.114%, n = 6) (P≤0.001 in all cases). On water immersed SiO_2_, average overnight nitrogen levels (0.258%±0.258%, n = 5) were also different from levels of week 1 (9.242%±0.206%, n = 5), week 2 (9.158%±0.474%, n = 4) and week 3 (9.513%±0.607%, n = 4) (P≤0.001 in all cases). Finally, on horse serum immersed SiO_2_, differences between overnight nitrogen levels (13.159%±0.277%, n = 8) and levels of week 1 (9.166%±0.722%, n = 7) week 2 (8.646%±0.258%, n = 9) and week 3 (8.683%±0.270%, n = 6) were statistically significant (P≤0.001 in all cases). Differences in nitrogen levels between week 1, week 2 and week 3 in control and horse serum samples of parylene-C and SiO_2_ were never statistically significant. Conversely, differences between control and horse serum samples within each week were statistically significant in Parylene-C (P≤0.001) but not in SiO_2_ (P = 0.2). Figure 8 summarizes the development of nitrogen levels on all surfaces, over the course of 3 weeks. In serum immersed surfaces, nitrogen levels peak right after immersion, then gradually decrease to a plateau during the next 3 weeks. Nitrogen levels for water immersed substrates develop from a different starting point, but demonstrate a similar behaviour. On the Parylene-C however, nitrogen levels of water immersed samples are distinctly different from those of serum immersed surfaces.

In order to investigate whether adsorbed proteins can be eluted from the two substrates, we incubated parylene-C and SiO_2_ uniform surfaces in horse serum overnight (some surfaces from each substrate were only immersed in distilled sterile water (No HS controls)). We then rinsed the surfaces with SDS and scanned them with XPS.

**Figure 9:**
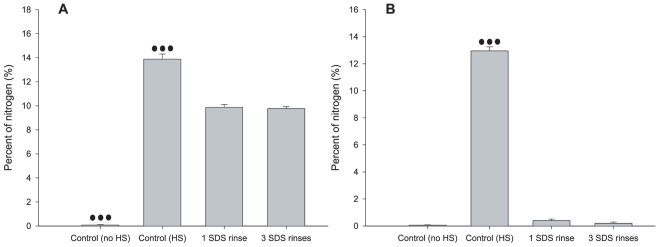
Nitrogen levels on serum immersed parylene-C and SiO_2_ after 1 or 3 SDS rinses. (A) Parylene-C. (B) SiO_2_. The first bar in both graphs denotes nitrogen levels in control samples that were immersed only in distilled sterile water, while the second bar is associated with control samples immersed in horse serum. Nitrogen is easily removed from serum immersed SiO_2_ substrates after 1 or 3 SDS rinses. On the other hand, significant nitrogen levels remain on serum immersed parylene-C surfaces even after 3 rinses with SDS. Three dots represent statistical significance of P≤0.001.

A one way ANOVA test on the nitrogen levels on Parylene-C revealed a statistically significant difference between average nitrogen levels of all pair wise combinations of control groups (No HS: 0.08%±0.031%, n = 3), (HS: 13.89%±0.421%, n = 3) and experimental groups (1 SDS rinse: 9.877%±0.228%, n = 3), (3 SDS rinses: 9.776%±0.159%, n = 5), (P≤0.001). However, differences between average nitrogen levels of the two experimental groups were not statistically significant (P = 0.988). A one way ANOVA test on the nitrogen levels on SiO_2_ revealed that only one control group (HS: 12.953%±0.298%, n = 3) had statistically different nitrogen levels from the other control (No HS: 0.063%±0.024%, n = 3) and experimental groups (1 SDS rinse: 0.4%±0.108%, n = 3), (3 SDS rinses: 0.208%±0.082%, n = 5), (P≤0.001). Differences between average nitrogen levels of other groups were not statistically significant (P = 0.884). The bar graphs of Figure 9 summarize these results.

## Discussion

Our results show that neuronal and glial conformity gradually deteriorates after the first week *in vitro*. The culture images suggest that glial cells first extent processes to the SiO_2_, as they are less sensitive to surface chemical cues than neurons [6]. It is likely that glia gradually deposit proteins onto the SiO_2_ substrate and form a “cellular carpet” onto which the neurons may subsequently migrate. This deposition of proteins from hippocampal cells and the degradation of the cytophobic background has been reported in other cell patterning studies as well [24]. A common response to this problem is the modification of the non-permissive background, so that it becomes resistant to protein adsorption. However, molecules, such as PEG, that are usually grafted onto the surface degrade over time. In addition, the majority of PEG-based cell patterning methods examine neuronal or cardiomyocyte compliance [31], [32]. Contrary to neurons and cardiomyocytes, glia divide in vitro and are also less likely to follow chemical cues. Therefore, glia are difficult to restrain on the patterns.

We suggest three explanations for the initial glial migration and the subsequent loss of cellular compliance to underlying Parylene-C stripes: i) the proteins responsible for glial and neuronal adhesion gradually desorb from the Parylene-C into the media, ii) proteins in the media regulating cell adhesion adsorb onto the SiO_2_ and progressively exceed those on Parylene-C, iii) glial mitosis encourages glial migration, which subsequently distracts neurons from their initial positions. The proliferation of glia in the cultures is evident in the gradual increase of nuclear density over the three weeks. As Figure 3d illustrates cellular division decelerates after the 2^nd^ week, which could be due to a membrane mediated neuronal inhibition [33] or glial contact inhibition. On the other hand, the XPS data clearly indicate that proteins are still present on the Parylene-C during the second and third weeks. Furthermore, nitrogen levels on serum and water immersed Parylene-C samples remain constant at different levels (Figure 8). This suggests that the protein profile on the Parylene-C serum immersed substrate is different from that of water immersed samples and does not change significantly over the 3 weeks. Similarly, nitrogen levels on SiO_2_ remain stable over 3 weeks (Figure 8). Finally, Figure 9a illustrates that even SDS (a surfactant often used for the elution and preparation of proteins in gel electrophoresis) cannot reduce nitrogen presence below 10%, which coincidentally is the level at which nitrogen stabilizes in serum immersed samples of Parylene-C. We therefore conclude that serum proteins do not just passively adsorb onto the parylene-C, but rather bind through specific hydrophobic interactions, that remain stable during a 3 week culture in media [20]. As a result, significant desorption of proteins from Parylene-C into the media is highly unlikely. Likewise, significant adsorption of proteins from the media onto the SiO_2_ beyond the first week is not supported by the XPS data. Parylene-C and SiO_2_ nitrogen levels are both close to 10% from the start of week1 to the end of week 3, with SiO_2_ nitrogen levels remaining constantly below Parylene-C levels. While we cannot exclude the possibility of protein exchange or conformational change on the two substrates based on the data, we believe it is more probable that the loss of glial and neuronal conformity to fabricated patterns is due to glia division.

It is attractive to consider glia and neurons as two competing patterning systems, as both glia and neurons fight for the same space on the patterns. Researchers have tried to overcome this issue by opting for high purity neuronal cultures, which do not contain any glia. An example of this are cultures of embryonic neurons, which are derived from rat embryos at E18, when glia have not yet emerged in the developing brain [34], [35]. Interestingly, in patterned cultures of embryonic cells, neurons first migrate toward cytophilic areas and after 2 weeks *in vitro* glia co-localize in the same regions [36]. This is probably due to the late emergence of glia in these cultures, which initially consist primarily of neurons. However, even in embryonic cultures the colocalization of glia and neurons decreases from 90% to 80% over the course of culture, due to glial proliferation [36]. Additionally, in embryonic cultures it is often difficult to preserve the neuronal population without glial support, while spontaneous neuronal activity becomes significant only after the emergence or addition of a glial population [34]. It is therefore more productive to view the glial-neuronal patterning system as symbiotic rather than competitive. By controlling the seeding cell density, the researcher can ensure there is enough space on the patterns initially to accommodate both cell types. If glia and neurons were competing at this stage, only one of these two cell types would be patterned. In our case however, until the end of the first week of culture, both glia and neurons remain on the Parylene-C patterns. It is uncontrolled mitosis that forces glia off the patterns and leads to the emergence of unpatterned neurons. Consequently, in a glial-neuronal patterned co-culture, glia will dictate neuronal patterning fate, by directing neurons to locales of dense glial proliferation. Mutual conformity to the patterns can be engineered for both cell types, by incorporating a moderating mechanism for glial division into the patterning technique.

Glial mitosis is often moderated with the addition of AraC in the media [25], [37]. In this study, we introduced small doses of AraC into the media after the first week, but interrupted its effect every 2 days, by exchanging the media. We did this in an attempt to mitigate the deleterious side effects of AraC to neurons [38], [39]. Unfortunately, Figures 6a and 6d reveal that this intermittent treatment was not adequate in preventing neuronal apoptosis. Nonetheless, this treatment reduced glial division and preserved cellular conformity as the bargraph of Figure 5 illustrates. The culture image of Figure 4b reveals that in the AraC treated cultures there is no glial carpet after 21 DIV, as in the controls (Figure 2c).

We used BDNF, which is a well known neuro-protective [40] and neuronal growth enhancing agent [41], [42] to mitigate the cytotoxicity of AraC. In a similar study Hardelauf et al. [43] highlight the protective effect of BDNF against acrylamide induced neurite degeneration in patterned cultures. In our study, the images of Figure 6 reveal the beneficial effect of BDNF on the neuronal population of cultures treated with AraC. At 2 weeks in vitro (Figures 6b and 6c) even a small dose of BDNF (10 ng/ml) is enough to preserve the neuronal population. During the third week however (Figures 6e and 6f), a larger dose (20 ng/ml) is necessary. The BDNF treatment has an effect in the conformity of both neurons and glia as illustrated in the bargraph of Figure 7. CGI and NeCI drop as the BDNF dose increases at 14 and 21 DIV. To our knowledge, our study is the first to investigate the concurrent application of AraC and BDNF on patterned cell cultures.

The statistical analysis of the conformity indexes did not reveal any significant differences between stripe patterns of different widths. This result agrees with our previous work [19] and reinforces our argument that the combination of a serum treated Parylene-C/SiO_2_ cell patterning substrate provides a strong guiding signal to cultured cells.

### Conclusions

In this study we examined glial and neuronal conformity to patterns of Parylene-C over the course of 3 weeks. Initially, we documented a gradual loss of cellular conformity, which we believe is primarily due to glial mitosis. Characterization of the Parylene-C surface reveals that proteins are constantly present over the culture period, as nitrogen levels remain stable. The addition of Ara-C in the media mitigates glial division and preserves cellular compliance to underlying patterns. Neuronal apoptosis on cultures treated with Ara-C can be addressed with the introduction of BDNF, which enhances neuronal growth. We believe that the Parylene-C patterning technique in combination with an optimally controlled glial population will permit neuronal cultures of high topological precision over long time periods. These cultures can be of significant value to the neuroengineering and neuroscientific communities, as they can be interfaced to MEAs via capacitive coupling, to record activity in patterned neural networks.
